# Ultrasound in the Food Industry: Mechanisms and Applications for Non-Invasive Texture and Quality Analysis

**DOI:** 10.3390/foods14122057

**Published:** 2025-06-11

**Authors:** Nama Yaa Akyea Prempeh, Xorlali Nunekpeku, Arul Murugesan, Huanhuan Li

**Affiliations:** School of Food and Biological Engineering, Jiangsu University, Zhenjiang 212013, China; 5102220318@stmail.ujs.edu.cn (N.Y.A.P.); 5102230336@stmail.ujs.edu.cn (X.N.); chemarul91@gmail.com (A.M.)

**Keywords:** ultrasound technology, food quality evaluation, non-destructive testing, textural properties, acoustic cavitation

## Abstract

Ultrasound technology has emerged as a transformative tool in modern food science, offering non-destructive, real-time assessment and enhancement of food quality attributes. This review systematically explores the fundamental mechanisms by which ultrasound interacts with food matrices, including mechanical effects such as acoustic cavitation, localized shear forces, and microstreaming, as well as thermal and acoustic attenuation phenomena. Applications of ultrasound in food texture evaluation are discussed across multiple sectors, with particular emphasis on its role in assessing moisture distribution, fat content, structural integrity, and microstructural alterations in meat, dairy, fruits, and vegetables. The versatility of ultrasound—spanning low-intensity quality assessments to high-intensity processing interventions—makes it an invaluable technology for both quality control and product innovation. Moreover, emerging innovations such as ultrasound-assisted extraction, non-thermal pasteurization, and real-time quality monitoring are highlighted, demonstrating the synergy between ultrasound and advanced technologies like AI-driven data interpretation and portable, handheld sensing devices. Despite these advances, challenges related to technical limitations in heterogeneous food systems, high initial investment costs, scalability, and the absence of standardized protocols remain critical barriers to widespread adoption. The future directions emphasize the integration of ultrasound with multi-modal approaches, the development of miniaturized and cost-effective equipment, and the establishment of global regulatory standards to facilitate its broader application. Overall, ultrasound is positioned as a key enabler for sustainable, efficient, and non-invasive quality assurance across the global food industry.

## 1. Introduction

The modern food industry is under increasing obligations to provide graded, nutritious, and minimally processed products in addition to the standard of attributes sought by consumers. These expectations include the demand for freshness, natural formulations, and minimal chemical additives, culminating in a need for cutting-edge sustainable processing technologies [1,2]. In this scenario, food texture and quality verification has perhaps become the most crucial consideration for food manufacturers, regulators, and researchers. Texture, especially, not only serves as a key determination of sensory acceptance, but it is also an important marker of processing functionality, shelf life, and food authenticity [3,4].

Ultrasound involves the transmission of sound waves above 20 kHz—beyond the upper limit of human hearing—with high-frequency ultrasound typically ranging from 1 MHz to 100 MHz, especially used in imaging and quality sensing applications. It is capable of inducing mechanical effects (e.g., acoustic cavitation and microstreaming), which can profoundly impact the mass transfer, protein restructuring, emulsification, and tenderizing processes. These acoustic factors, such as wave velocity, attenuation, reflection, and scattering, react positively to changes in composition and structure (i.e., moisture content, fat structure, protein conformation, and cellular structure), all of which can be quantified and reported through ultrasound measurements. Importantly, it enables in situ and real-time assessment without affecting the food item, which fits harmoniously with the industry’s growing movement towards automation, traceability, and sustainability [5,6]. The ways in which ultrasound enables criteria to be changed divide it from other food products because few ingredients can change solubility and dispersion of solid matter in water without the use of excessive heat or chemical manipulation that have clean label and energy conservation properties [7]. Recent advancements in ultrasound-based emulsification, particularly in combination with protein–polysaccharide complexes, have shown immense potential. As highlighted in Qayum et al. [8], ultrasound facilitates the formation of fine, stable emulsions by reducing droplet size and modifying protein conformation via acoustic cavitation. These changes enhance rheological and emulsifying properties thereby enabling the design of sustainable, eco-friendly, and health promoting colloidal systems for foods. Such emulsions stabilized by natural biopolymer complexes hold special significance for industries pursuing clean label, functional formations across dairy, meat, and beverage sectors.

In the literature, ultrasound has been extensively evaluated and shown its usefulness across a variety of food sectors. For example, in the meat sector, ultrasound has been compared to control processing to improve tenderness, offer increased marination absorption, improve water-holding capacity in meat products, and improve emulsion stability [9,10]. Yeung and Huang [11] have demonstrated using ultrasound and aged samples have improved texture characteristics with associated subjective acceptance scores. Dairy products made using ultrasound have been shown to have increased homogenization efficiency, increased yogurt viscosity (steadiness of yogurt during production), and increased cheese yield through protein modification [12]. For fruits and vegetables, ultrasound pretreatments have improved the retention of initial food firmness, reduced enzymatic browning, and improved behavior during drying processing [13].

Additionally, other applications of ultrasound have been incorporated into new manufacturing methods, such as decreased oil uptake for fried foods [14] and speeding up fermentation and aging processes for fermented foods [15,16]. These examples demonstrate that ultrasound can be multi-faceted: it can improve texture and the formulation of healthier products while utilizing less energy in food processing. As previously mentioned, the existing literature regarding ultrasound still remains disjointed. Many studies investigate particular functions or types of food, lacking a comprehensive understanding of the ways ultrasound impacts structure and texture in varying matrices [17]. Operational challenges, such as process optimization, scale-up, and standardization, are frequently neglected. Broader challenges such as an optimal frequency and exposure time of ultrasound, food composition, and unwanted occurrence (ex., protein denaturation or lipid oxidation) must be explored [18]. Therefore, a review that brings together existing knowledge on ultrasound to improve food quality and texture, while discussing operational issues and possible future avenues, is warranted.

This review aims to bridge these gaps by presenting a targeted and integrative evaluation of ultrasound technology as a quality improvement tool, inclusive of textural improvements. We explore the underlying mechanisms and applications related to ultrasound technology across various products, including meat, dairy, seafood, fruits, vegetables, and processed foods. We also identify operational challenges and consider future opportunities, including use with emerging technologies—such as artificial intelligence—and other advanced manufacturing processes. Ultimately, by synthesizing our current knowledge and indicating opportunities for innovation, we aim to provide guidance to researchers, industry representatives, and technologists on how to use ultrasound on their own consumer-driven, sustainable, and efficient food processing initiatives.

## 2. Fundamentals of Ultrasound Technology

### 2.1. Principles of Ultrasound in Food Quality Enhancement

**Figure 1:**
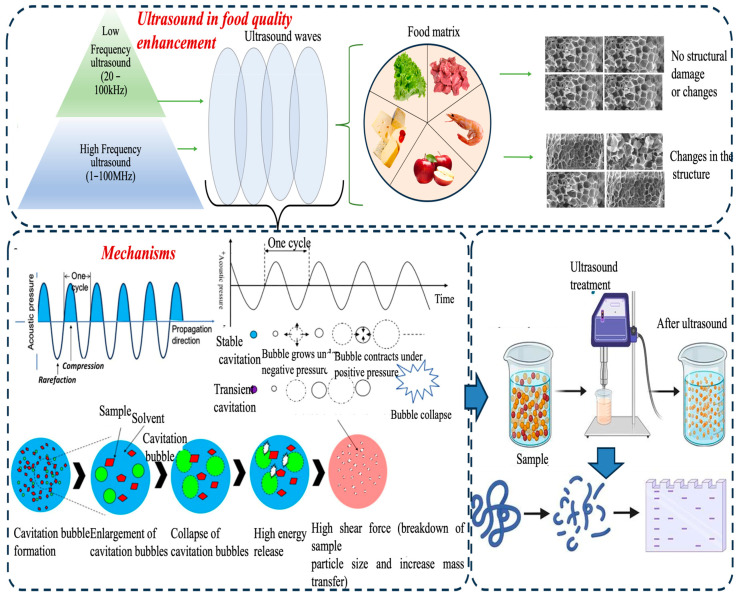
Overview of ultrasound mechanisms and effects in food quality enhancement. (**A**) Classification of ultrasound by frequency and its effects on various food matrices, showing outcomes such as no structural damage or induced microstructural changes depending on treatment parameters; (**B**) Schematic representation of ultrasound cavitation mechanisms, including stable and transient cavitation, bubble dynamics, and the resulting high shear forces that contribute to structural modifications and mass transfer improvements; (**C**) Illustration of ultrasound treatment setup and its impact on food samples, leading to the breakdown of macrostructures into finer elements, enhancing diffusion, dispersion, and functional properties.

Ultrasound technology, defined by sound waves above 20 kHz, enhances food quality through acoustic cavitation and microstreaming [2,9]. Cavitation involves the rapid formation and collapse of bubbles (Figure 1), generating localized shear forces and microjets that drive changes in food matrices [19]. For instance, ultrasound-mediated cavitation is known to weaken muscle fibers which leads to a tenderizing effect in meat products and improved infiltration of marinade [11]. Acoustic streaming and microstreaming further support cavitation by promoting fluid motion, accelerating diffusion, and increasing efficiency in processes like extraction, dehydration, infusion, and crystallization [12]. These benefits extend to dairy, where ultrasound assists in homogenization and gelation; seafood, by improving texture and potentially aiding microbial reduction; and fruits and vegetables, by enhancing drying, preserving firmness, and reducing enzymatic browning. In processed foods, ultrasound supports emulsification and structural stability—key for improving quality and shelf life [20]. Processing efficiency depends on factors such as frequency, intensity, exposure time, and the food matrix’s physicochemical properties (e.g., composition, density, elasticity). Most food applications utilize high-intensity, low-frequency ultrasound (20–100 kHz) for structural modification, whereas low-intensity ultrasound is used for non-destructive quality assessments. High-frequency ultrasound (1–100 MHz) is preferred for imaging, sensing, and non-destructive testing due to its superior resolution [18]. While ultrasound has distinct advantages (reduced processing time, energy consumption, thermal damage, and improved sensory attributes), there are also issues associated with its use. The food matrix is considerably heterogeneous and food structures typically contain multiple phases (e.g., fat, water, air), with structural composition affecting ultrasound propagation and cavitation efficiency [21]. Though ultrasound reduces processing time and energy use, excessive intensity can induce unwanted protein denaturation or oxidation. Continued advancements in equipment design, parameter control, and hybrid processing are expanding its role in texture optimization and shelf life extension across meat, dairy, seafood, fruit, vegetable, and processed food sectors.

### 2.2. Ultrasound Application Techniques in Food Quality Enhancement

Ultrasound technology offers a range of application techniques tailored to food processing objectives, such as enhancing texture, accelerating mass transfer, and modifying structural properties [9]. In practice, ultrasound is applied via direct sonication, using probe systems for focused, high-energy delivery, or indirect sonication, where ultrasound baths or flow systems provide uniform energy dispersion suitable for fragile or large-volume products [22]. Advanced techniques like multi-frequency ultrasound enhance cavitation efficiency and treatment uniformity, benefiting applications such as freezing, drying, or extraction [23]. High-intensity methods have been developed for marination rates and tenderization of meat products [24,25] whereas indirect methods can be used for crystallization control or improved drying kinetics in fruits and vegetables [4,26]. Accommodating integration into continuous processing lines brought scalability by decreasing processing times and increasing uniformity of the product [26]. In addition, techniques such as pulse-echo, through-transmission, and scattering-based approaches allow for in situ observations of compositional and structural parameters. Dong et al. [27] used scattering-based ultrasound to evaluate particulate distribution and stability of a food emulsion while Dourado et al. [28] used pulse-echo and through-transmission to evaluate textural properties in restructured meat, and the results were strongly correlated with traditional textural evaluation. Selecting the appropriate ultrasound technique—based on intensity, mode, and frequency—is critical for optimizing processing efficiency and ensuring desired quality outcomes across diverse food systems.

### 2.3. Instrumentation Design and Process Optimization

The efficiency and reliability of ultrasound-assisted food processing depend on advanced instrumentation design and precise control of operational parameters. Modern systems employ various configurations, from probe-type devices to flow-cell reactors, each tailored to specific processing needs [22]. Innovations such as multi-frequency transducers and automated control systems have enhanced consistency by enabling dynamic adjustment of cavitation intensity [24,29]. Key operational parameters—such as frequency, intensity (amplitude), treatment duration, and temperature—directly influence cavitation dynamics, shear forces, and microstreaming effects. For example, lower frequencies (20–40 kHz) generate more aggressive cavitation suitable for disrupting muscle fibers, while higher frequencies are preferred for delicate modifications like emulsification or gel stabilization [26]. The interaction between these parameters and food characteristics (e.g., moisture content, viscosity, geometry) necessitates careful process optimization. Zhang et al. [30] indicated that by manipulating the frequency of the transducer and coupling conditions, they were able to enhance the gelation behavior of dairy products and improve textural quality with no structural damage or defects. Görgülü et al. [31] also demonstrated that, by modifying scanning speed and amplitude, moisture control of baked goods resulted in improved consistency and less quality loss during processing. Additionally, studies have shown that ultrasound treatment can significantly alter the structural properties of proteins, enhancing their functional characteristics [20].

However, heterogeneous matrices can become inconsistent with cavitation and porous foods can attenuate ultrasound energy, while also having the potential risk of overprocessing [26]. The main approach is to use real-time monitoring/control systems for ultrasonic energy [24] and other hybrid forms of treatment, especially manosonication (ultrasound + pressure), to allow enhanced monitoring and controlling of the processing dynamics [32]. In general, adapting the instrumentation and refining the processing parameters is key to achieving practical and efficient systems that can capitalize on ultrasound as a tool to improve food texture, structure, and quality in diverse industrial-scale applications.

## 3. Mechanisms of Ultrasound Interaction with Food Matrices

Ultrasound technology modifies food matrices due to mechanical, acoustic, and thermal effects that modify structural, textural, and functional properties. The interactions (wave propagation, energy transfer, and cavitation) are essential for process efficiency and for processing food to improve quality [33]. In this section, we describe these mechanisms and how they are used for food modification, along with related challenges.

### 3.1. Mechanical Effects of Ultrasound on Food Matrices

**Figure 2:**
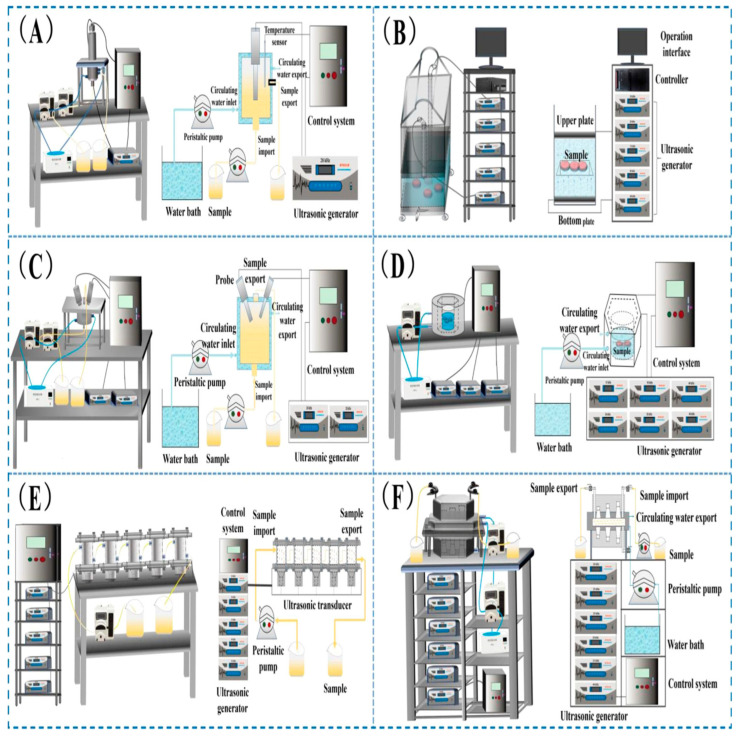
Schematic representation of various ultrasound-assisted systems used in food matrix processing. Systems include (**A**) Cup-type single-frequency counter-current ultrasonic device; (**B**) Dual-frequency sweeping ultrasonic device; (**C**) Cup-type dual-frequency counter-current ultrasonic device; (**D**) Hexagonal tri-frequency ultrasonic device; (**E**) Cylindrical five-frequency counter-current ultrasonic device; (**F**) Slit-type six-frequency ultrasonic device, each integrated with control units, pumps, and thermal regulators to modulate cavitation and acoustic effects under different conditions. Reproduced with permission from [2].

The mechanical effects of ultrasound are primarily due to acoustic cavitation—the rapid formation, growth, and implosion of sub-microscopic bubbles in liquids or semi-liquids. When these bubbles implode, they release extremely strong shockwaves, microjets, and shear forces that cause localized structural damage. Mechanical effects are especially important in modifying the microstructure of food, especially cellular matrices like meats, fruits, and vegetables [34]. At low intensities, ultrasound plays a non-invasive role and is used as a gentle method for probing structural properties. At high intensities, cavitation causes breaks in cell walls and cell membranes and enhances processes that lead to tenderization, extraction, and emulsification. For instance, several experimental configurations for ultrasound-assisted food processing—ranging from bath setups to probe and multi-channel systems—are used to deliver controlled cavitational energy and fluid flow under diverse conditions (Figure 2). Bernardo et al. [35] indicated that ultrasound-assisted tenderization aided in the degradation of collagen networks in pork which consequently improved texture and moisture retention. Umair et al. demonstrated enhanced extraction and bioactivity of phenolic compounds from carrot pulp when subjected to ultrasound-assisted extraction prior to boiling, yielding high antioxidant values [36], an effect that aligns with recent enzymatic protein hydrolysis by Qian et al. [37]. In protein-rich foods, mechanical forces partially denature proteins, exposing reactive sites that improve water-holding capacity, gelation, and emulsification properties—key factors in applications like homogenization and textural modification [38,39,40]. Altered structures can enhance sensory qualities, reduce the energy or time to change the structure, improve nutrient bioavailability, and increase consumer acceptance. Nonetheless, ultrasound enhances product attributes within a narrow framework of conditions that need to be carefully manipulated as exposure time or mismanaged conditions can lead to overprocessing or capitalize on beneficial sensory attributes to the point they damage fragile structures or degrade sensitive nutrients. Also, stoichiometric deviation in cavitation behavior with respect to both time and severity due to matrix heterogeneity has a potential detrimental effect on reproducibility.

In summary, ultrasound’s mechanical effects, driven by cavitation-induced forces, offer versatile solutions for modifying food structure and texture. When properly controlled, these mechanisms enhance processing efficiency and product quality. However, balancing intensity and exposure time is essential to maximize benefits while avoiding detrimental effects.

### 3.2. Acoustic and Thermal Effects

Ultrasound not only causes mechanical disruption but also has its own acoustic and local thermal effects in food that can impact both structure and functionality. Unlike traditional heating, localized thermal energy produced by ultrasound is induced by the collapse of cavitation bubbles leading to short-lived and transient regions of high temperature and high pressure. This energy is extremely localized and transient but represents a source of energy that can create significant microstructural changes, including protein denaturation, lipid reorganizations, and increased gel formation without actually changing the bulk temperature of the food matrix [41]. The advantages of these local thermal effects are particularly advantageous for maintaining heat-sensitive nutrients including vitamins and antioxidants, as ultrasound is a useable non-thermal processing method. For example, Wang et al. demonstrated, through using controlled ultrasound heating, that the gelation and emulsification properties of plant-based proteins were enhanced, without affecting nutritional value [42]. Likewise, Juliano et al. [43] found that using ultrasound allowed for low-fat dairy emulsions to be stabilized due to the better dispersion of fat globules being aided by mild heating and the acoustic scattering effects. More recent studies explain these phenomena. Yang et al. [44] investigated the effects of ultrasound pretreatment on the Maillard reaction of grass carp protein hydrolysates, revealing that ultrasound enhanced protein unfolding and aggregation, leading to improved flavor and functional properties. Additionally, Qian et al. [37] reviewed the mechanisms of ultrasound-assisted enzymatic protein hydrolysis, highlighting how ultrasonic pretreatment can modify enzyme and substrate structures, thereby enhancing hydrolysis efficiency and the bioactivity of resulting peptides. In addition to the thermal effects, the acoustic aspect can be described through the energy attenuation when sonic waves pass through the food matrix, which has a wide applicability to agri-food science. Energy is absorbed and/or scattered depending on the acoustic properties of the food and is related to the structural attributes of the food affecting viscosity, phase behavior, particle distribution, and other attributes related to processes such as emulsification and the stabilization of dispersions [45]. Acoustic energy transfer can be employed to manipulate these properties intentionally and make directed changes in food texture and stability. One important chemical consequence of ultrasound is the generation of free radicals through acoustic cavitation. The collapse of microbubbles produces localized extremes up to 5000 K and 1000 atm that split water molecules into reactive oxygen species (ROS) like hydroxyl and hydrogen radicals. These ROS can oxidize lipids, degrade bioactive compounds, and alter amino acids, impacting food structure, flavor, and nutrient stability [46]. They also contribute to microbial inactivation. While beneficial for safety, these effects must be carefully managed to avoid unintended degradation. An important distinction is that the action of the two (acoustic and heat) is twofold and needs to be monitored closely. In other words, injecting too much energy into the system could have unintended consequences, such as overdenaturation, oxidation, or destroying the fragile properties of the material. Thus, ultrasound parameters need to be optimized (with respect to frequency, intensity, and treatment time) to maintain the structural features while maintaining stability of the food product. The combined effects of the acoustic potential of ultrasound, as well as the localized thermal potential, provide one of the few adaptable methods to modify food structure, enhance functional properties, or maintain health benefits while providing controlled applications of energy. This energy provides opportunities to develop emulsions, restructure protein, and optimize texture for modern processed foods and highlights ultrasound as a valuable, non-heat technology in the future of food processes.

### 3.3. Correlation of Acoustic Properties with Texture

Ultrasound technology provides a non-destructive approach to evaluate food structure when relating several acoustic properties: velocity, attenuation, and reflection. Each of these properties has been previously correlated with a textural attribute: firmness and elasticity, for example, were associated with a good correlation with velocity; as was cohesiveness with attenuation; and finally, reflection can be captured with a single measurement to obtain information about layers or structure regarding a food product (e.g., gel-like foods or processed foods). Each of these structural attributes can only be determined once a product has been evaluated with ultrasound variables validated with texture attributes. For example, there is a strong correlation with the velocity of a material and the prediction of meat tenderness because generally high velocity equals tougher textures of meat; specifically, Cheng et al. [41] showed that the velocity predicted firmness of dairy gels, determining that ultrasound could be used to measure texture attributes quickly and non-destructively in food products. Similarly, Guillermic et al. [47] confirmed predictions of both moisture content and structural consistency in plant-based meats appearing in an attenuation pattern. Supporting this, Zhang et al. [48] reported that ultrasound velocity showed strong correlation with elastic modulus and toughness in wheat gluten structures. Furthermore, Wang et al. [40] observed that attenuation coefficients varied significantly with changes in protein conformation during gelation, allowing precise tracking of textural transitions.

However, challenges remain. These include the variability of the matrix, temperature effects, and additional complexity in multi-phase systems (e.g., emulsions or fibrous products) that can all affect the measurement accuracy. Furthermore, while acoustic analysis of food products with an irregular structure can be used, the acoustic data must be calibrated carefully and verified to be a valuable tool in analysis. Overall, ultrasound texture analysis provides advantages by usage intensity in destructive mechanical testing, besides offering faster and repeated outcomes, while enhancing quality control on a production level. Structuring acoustic responses to sensory-relevant properties, such as mouthfeel and firmness, further establishes ultrasound in product development and ensures consistency in outputs across a variety of products (including meat, dairy, and plant-based foods).

## 4. Ultrasound for Comprehensive Quality Assessments

Ultrasound technology has become an essential tool for non-destructive, real-time quality assessment in the food industry. By analyzing acoustic responses, ultrasound enables precise monitoring of key attributes such as moisture content, fat distribution, and structural integrity—factors critical to product consistency, shelf life, and consumer satisfaction. Its ability to detect internal changes without altering the product makes it invaluable for continuous quality control across diverse food systems. This section explores how ultrasound is applied to evaluate these core parameters, emphasizing both its practical benefits and operational challenges.

### 4.1. Moisture Content and Water Distribution

Proper control of moisture content and water distribution are key factors in food texture, yield, and shelf life [49]. Ultrasound is quick approach to observing the moisture content and water distribution in processed foods by utilizing the sensitivity of acoustic wave velocity and attenuation to water in food matrices [50]. Differences between free and bound water can be explored through changes in wave speed and/or energy loss, which can be translated into the hydration status and water-holding capacity of processed foods. In the complex preparation of meat products, ultrasound has been directly applied to monitor moisture retention during marinating, storage, and problem-solving cooking processes to ensure the products maintain juiciness and lose less weight [25]. Wu et al. [51] were able to show that ultrasound velocity measurements had a similar correlation to both water content and water mobility in pork as accurate traditional gravimetric water content methods. Jiang et al. [52] used ultrasound imaging for the postharvest management of fruits as a methodology to monitor moisture migration in apples during dehydration. This permitted greater control over fruit texture and shelf life. A study by Wang et al. [53] investigated the effects of ultrasound-assisted sous-vide cooking on spiced beef, revealing that ultrasound treatment enhanced water retention and improved textural qualities by maintaining lower cooking loss and preserving muscle fiber integrity. Additionally, Xu et al. [54] examined the impact of ultrasound immersion freezing on red radish, demonstrating that ultrasound treatment led to more uniform water distribution and better preservation of quality attributes during freezing. Although ultrasound is a very effective tool, ultrasound accuracy can be reduced in foods with complicated structures, temperature variation, uneven distribution of water, etc. To gain the most accurate measurements possible, further calibration with existing standard reference methods is required especially in complex food matrices or when measuring fibrous products. All of these factors considered, ultrasound is a strong technology to help monitor moisture dynamics, improving quality control in industry sectors such as meat, fresh produce, and processed foods.

### 4.2. Fat Content and Distribution

The correct measurement of fat content and its distribution is vital for controlling the various aspects of the product’s texture, taste, and nutritional content in high-fat foods. The ultrasound can detect fat by determining the patterns of scattering and attenuation of ultrasound signals as they pass through fat globules, giving a quick and non-destructive measure of fat concentration or globule size and the uniformity of fat distribution. Ultrasound is most commonly employed in the dairy industry to monitor the efficiency of homogenization, which is needed to maintain stable emulsions such as cream, milk, and mayonnaise. Ultrasound provided results for fat globules and size distribution in mayonnaise, which showed a close correlation with the stability of the product released during storage [55]. Ultrasound has also been used very effectively in meat processing to determine the intramuscular fat (IMF) content in relation to marbling, which relates to the quality of meat. Fabbri et al. found a very strong relationship between ultrasound and IMF for predicting differences in IMF at levels established in traditional carcass grading [56]. However, in non-homogenized systems (e.g., butter) or very heterogeneous systems, properly analyzing samples may be challenging. For example, if a sample has an uneven fat distribution, this will be reflected in the ultrasound signal. Additional factors such as temperature and the sensitivity of the ultrasound measurement will also impact the accuracy of the measurement. Therefore, calibrating all ultrasound measurements against chemical analysis is necessary. In conclusion, while ultrasound may be less effective in heterogeneous samples, it is still a quick and non-invasive option for assessing fat content and distribution, which could be used for quality, grading, or consistency of dairy products, meats, and emulsions.

### 4.3. Structural and Microstructural Alterations

Ultrasound detection of structural and microstructural changes has wide-ranging applications in food processing [57] and storage by monitoring changes in acoustic impedance and wave scattering. Acoustic properties can be influenced by relatively minor internal changes, making ultrasound a promising method to monitor the real-time development of texture, gelation, and degradation processes. Therefore, ultrasound could be used for the real-time monitoring of texture and microstructural changes associated with various products [58]. For various meat products, ultrasound effectively follows structural changes associated with heating, for instance, protein denaturation and gel formation will enhance the textural and binding properties of a canned product [59]. Yan et al. [60] demonstrated that ultrasound-assisted processing of whey protein isolate–chitosan complexes significantly altered structural characteristics, improving binding and functional capacities. In dairy, ultrasound is therefore a valuable method of monitoring the extent of gel network formation in yogurt and cheese products as well as correlating acoustic signals to gel strength and consistency [61]. Similarly, Cheng et al. [62] found that divergent ultrasound pretreatment altered the hardness and microstructure of whey protein gels, influencing their digestibility and structural uniformity. Further, ultrasound identifies changes during storage that could indicate the first signs of deterioration that could affect product quality; for example, ultrasound detects fat coalescence phenomena in emulsified products or water migration phenomena in frozen products [63]. For instance, Li et al. [64] used ultrasound to monitor protein unfolding in meat emulsions exposed to heat treatment to understand texture optimization. Likewise, Akdeniz et al. [65] used ultrasound to identify the structural breakdown of frozen yogurt by monitoring scattering profile changes, permitting the early management of quality. Further, Xu et al. applied multi-frequency ultrasound to modify starch–polyphenol complexes, reporting significant changes in food matrix organization and digestion behavior [66]. In a related study, Xu et al. [67] used flat sweep and pulsed ultrasound to assess enzyme microstructure and activity in mushroom polyphenol oxidase, emphasizing ultrasound’s precision in structural evaluation. In summary, although ultrasound may be sensitive to changes, it may also be limited in applications where the matrix is too heterogeneous or opaque since there are many forms of scattering and signal absorption that could improve signal accuracy. Achieving reproducible results will require careful calibration and control over environmental variables, including temperature and moisture. In summary, ultrasound technology offers a holistic, non-destructive, and comprehensive approach to probe and measure critical quality parameters in food systems, such as moisture movement, fat dispersion, and structural integrity. Its real-time, rapid nature makes ultrasound technology valuable for quality control, limiting product and production waste, and ensuring consistency of products during processing and storage operations. Although matrix complexity and calibration challenges remain, the continued development of ultrasound instrumentation and data analytic techniques will give ultrasound a greater role in contemporary food manufacturing.

## 5. Applications of Ultrasound in Food Texture Evaluation

### 5.1. Applications in Fruits and Vegetables

**Figure 3:**
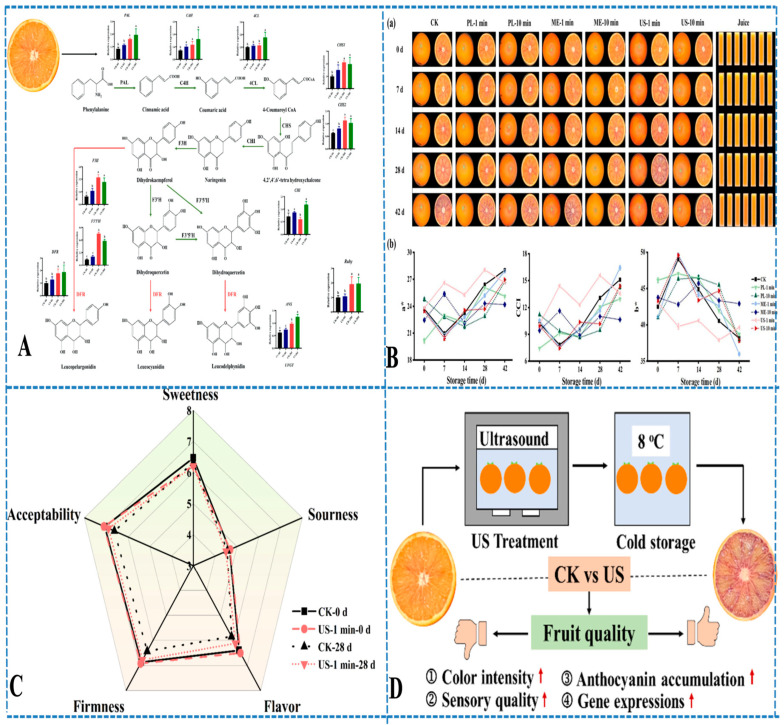
Effects of ultrasound treatment on postharvest blood oranges over varying storage durations. (**A**) Anthocyanin content in blood oranges treated with ultrasound for different durations; (**B**) (**a**) Visual assessment of orange slices and juices over 0–42 days of storage under different treatments, including control (CK), pulsed light (PL), microwave (ME), and ultrasound (US) for 1 and 10 min; (**b**) Quantitative changes in citrus surface color (a*), Citrus Color Index (CCI), and juice brightness (L*) during storage, illustrating improved color retention and visual quality under ultrasound treatment; (**C**) Radar chart comparing sensory attributes—sweetness, sourness, flavor, firmness, and acceptability—between control and ultrasound-treated fruits at day 0 and day 28; (**D**) Summary diagram showing that ultrasound treatment combined with cold storage (8 °C) enhances overall fruit quality through increased color intensity, sensory scores, anthocyanin accumulation, and gene expression compared to control (CK). Reproduced with permission [71].

Fresh fruits and vegetables are some of the most highly perishable items, so texture assessment and maintenance are critical to shelf life and quality [68,69]. Uses of ultrasound technology not only provide non-destructive, rapid information on textural properties but can also lead to efficiency in processing and the retention of nutrition. Among many interesting applications, salient applications would be in the arena of texture assessment that is non-invasive. Ultrasound is capable of providing information about attenuation and wave velocity, leading to predictions about both firmness and structural integrity. For example, in a study by Khuriyati et al. [70], they reported that ultrasound could accurately estimate melon firmness and showed a strong correlation (R^2^ = 0.763) of acoustic signals to textural properties. Chen et al. [71] reported a similar study in which a short ultrasound treatment of blood oranges during cold storage not only improved texture but also increased the anthocyanin content by almost 30%, demonstrating that ultrasound could effectively measure quality and improve it as well (Figure 3).

Beyond assessment, ultrasound plays a vital role in texture improvement, particularly through ultrasound-assisted osmotic dehydration (UAOD) [72]. UAOD enhances mass transfer and decreases drying times while helping structural integrity. This improvement is primarily due to acoustic cavitation, which induces microchannel formation in the tissue, disrupts cell walls, and reduces internal mass transfer resistance. For strawberries, UAOD using low-hygroscopicity sugars improved drying rates and texture quality. This finding was also reported in pomegranate arils and pineapple slices, wherein the use of ultrasound treatment improved water-loss rates and temporal retention of desirable texture. Xu et al. [73] further demonstrated that dual-frequency ultrasound pretreatment improved freeze-drying efficiency and structural preservation in strawberry slices. A similar multi-frequency ultrasound treatment enhanced both the drying rate and quality preservation in pineapple slices, especially phenolic retention and antioxidant activity [74]. In carrots, ultrasound-induced microstructural changes allowed for more efficient drying at lower temperatures while preserving key nutrients [75]. Gao et al. [76] studied ultrasound’s effect on carrot texture and demonstrated that pectin–cell wall interactions could be favorably altered through sonication, enhancing firmness and drying performance. Lastly, these findings, along with the findings from similar studies on strawberries, pomegranate arils, and pineapple, can be found in Table 1.

**Figure 4:**
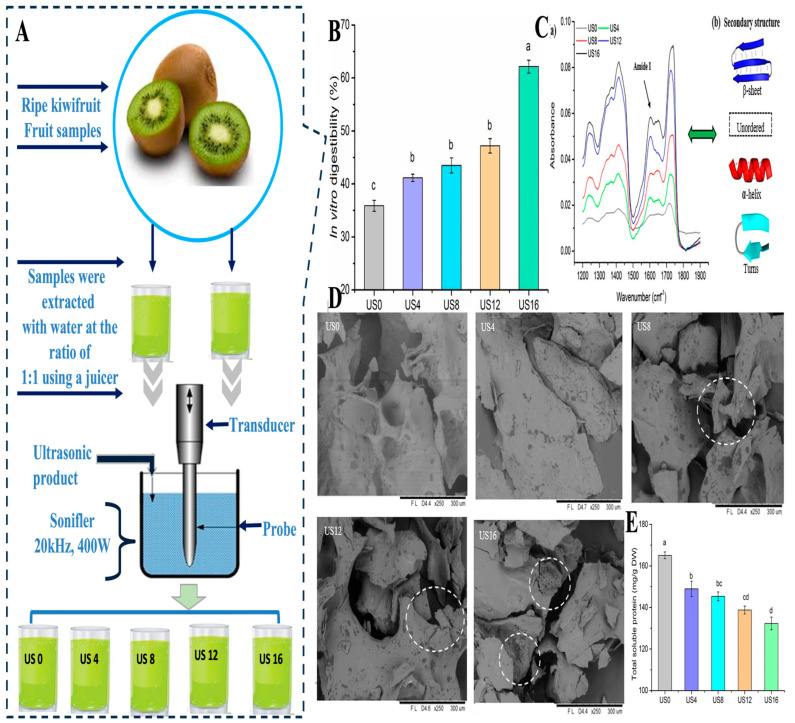
(**A**) Effects of ultrasound-assisted extraction on kiwifruit protein digestibility, structure, and microstructure. (**A**) Experimental procedure showing ultrasonic treatment of kiwifruit juice using a probe sonicator at 20 kHz and 400 W for varying durations (0–16 min); (**B**) In vitro digestibility (%) of kiwifruit proteins significantly increased with ultrasound treatment time, reaching the highest value at 16 min; (**C**) (**a**) FTIR spectra indicating changes in protein secondary structure (Amide I region) with increasing ultrasound time; (**b**) Diagram showing the associated structural motifs (α-helix, β-sheet, turns, and unordered regions); (**D**) SEM images showing progressive microstructural disruption of kiwifruit matrix under increasing ultrasound exposure (US0 to US16), with visible pore formation and fragmentation; (**E**) Total soluble protein content (mg/g DW) decreased as ultrasound time increased, suggesting protein aggregation or breakdown during sonication [106].

**Figure 5:**
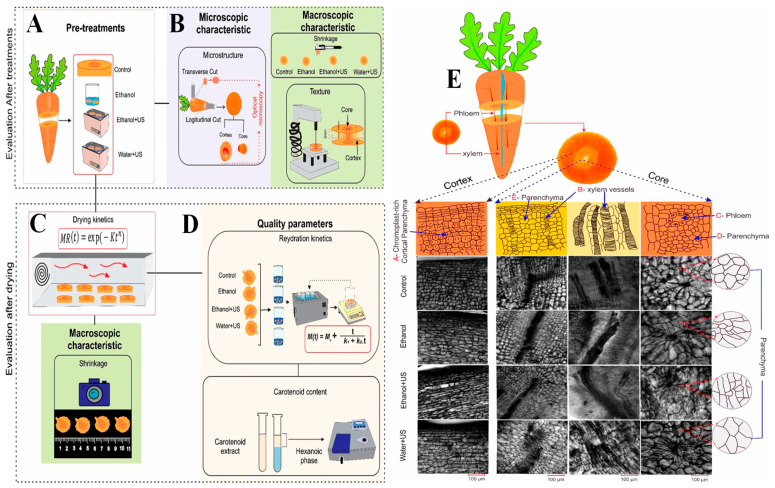
Schematic representation of the experimental setup and results from the study on ultrasound-enhanced heat pump drying (HPD) of carrots. (**A**) Pretreatment conditions (control, ethanol, ethanol + US, and water + US) and the subsequent evaluation of the microstructure using transverse and longitudinal cuts; (**B**) Changes in macroscopic characteristics, including shrinkage and texture, after drying; (**C**) Drying kinetics modeled using MR(t) = exp (−Kt^n^) to quantify moisture removal under various treatments, where MR(t) represents moisture ratio at time t, K is the drying rate constant, and n is the exponent reflecting the drying process; (**D**) Rehydration kinetics and carotenoid content analysis postdrying. (**E**) Microscopic images showing the changes in carrot tissue structure, including phloem, xylem, parenchyma, and cortical parenchyma, under different treatments. Adapted from [108].

A meta-analysis also showed that ultrasonic pretreatment increases hardness and quality of dried fruits and vegetables on a consistent basis [105]. Other benefits of ultrasound come from its antimicrobial properties, which are due to the free radical generation from cavitation and may lengthen the shelf life. Kiwifruit can trigger allergic reactions that can lead to death. As a result, Wang et al. [106] investigated the effect of high-intensity ultrasound (20 kHz, 400 W, 50% duty cycle) on the allergenicity, protein structure, and in vitro digestibility of kiwifruit proteins. The ultrasound treatment significantly disrupted the microstructure of kiwifruit tissues, leading to changes in the secondary structure of proteins, including a reduction in alpha-helixes and an increase in beta-sheet structures (Figure 4). They found that, after 16 min of treatment, the Act d 2 allergen content was reduced by 50%, and the in vitro digestibility of kiwifruit proteins improved from 35% to 62%. Additionally, the protein solubility decreased by 20%. Hong et al. [107] also confirmed that ultrasonic washing enhanced phenolic accumulation in red cabbage while improving microbial safety and storage stability. Also, Santos et al. [108] examined the effect of ultrasound-enhanced heat pump drying (HPD) on carrots with various pretreatments (control, ethanol, ethanol + US, and water + US). The study analyzed changes in the carrot’s microstructure, texture, and carotenoid content (Figure 5). Ultrasound treatment improved moisture migration and drying efficiency, with significant alterations in the microstructure and improved rehydration and nutrient retention. Xu et al. [109] showed that thermosonication of strawberry juice improved microbial stability, preserved volatiles, and minimized quality degradation compared to conventional thermal methods.

Overall, ultrasound is used as both a measurement and processing technique in fruit and vegetable applications. It is beneficial in the non-destructive analysis of texture, in addition to improving the quality of crop based on enhancing dehydration, maintaining nutrients, and controlling microbes. The flexibility of ultrasound makes it an effective modern additive in the produce process, which contributes to efficiencies, quality control, and consumer satisfaction.

### 5.2. Meat and Seafood Products

In the processing of meat and seafood products, texture evaluation is primarily centered on key attributes such as tenderness, muscle integrity, and the structural changes that occur during various processing stages. Among the emerging technologies, ultrasound has garnered considerable attention in both the meat and fish industries due to its ability to enhance product quality and processing efficiency. Ultrasound operates through a mechanism known as acoustic cavitation, which involves rapid pressure fluctuations that trigger physical and chemical changes within food matrices [110]. In meat processing specifically, ultrasound is employed to improve tenderness by disrupting muscle fibers and connective tissues, thereby resulting in a more desirable texture.

**Figure 6:**
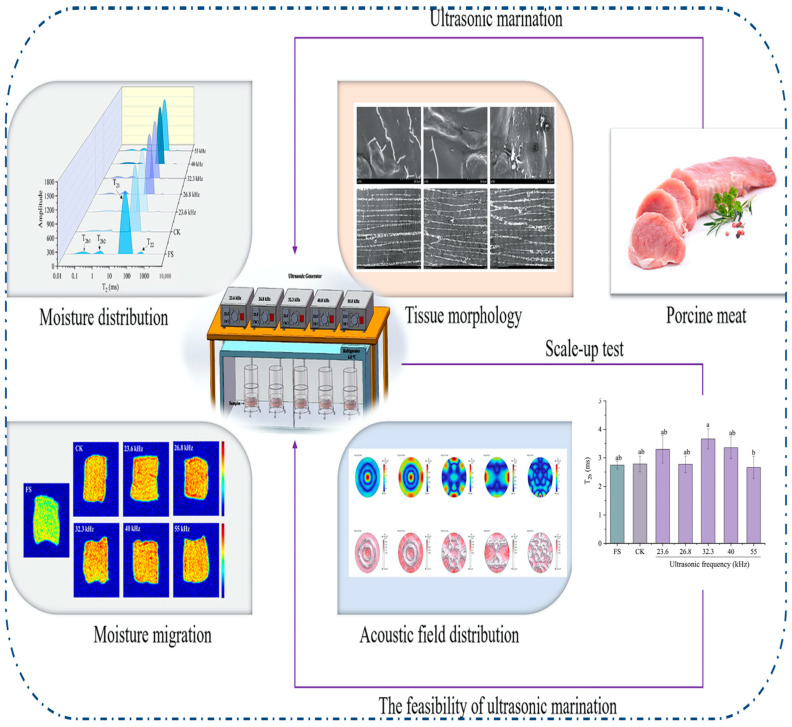
Effects of ultrasonic frequency on pork marination. Visualization includes moisture migration (MRI), acoustic field simulation, microstructural analysis (SEM), and salt penetration efficacy. Adapted from [10].

A detailed study by Guo et al. [10] on ultrasonic marination of pork provides a comprehensive illustration of how ultrasound affects meat quality through moisture dynamics and structural alterations. Using multiple ultrasonic frequencies (23.6 to 55 kHz), the study demonstrated changes in moisture migration, relaxation behavior (T_2_b, T_21_, T_22_), and tissue microstructure via NMR, MRI, and electron microscopy. As shown in Figure 6, ultrasonic treatment significantly enhanced water-holding capacity (WHC) by shifting free water to immobilized water and creating porous microstructures that facilitated NaCl diffusion. The most efficient marination occurred at 26.8 kHz due to optimal acoustic field distribution and minimal standing wave effects, as verified through COMSOL simulation (COMSOL Multiphysics version 5.5 was used). The study also confirmed scale-up feasibility in domestic refrigeration environments, underscoring the practicality of ultrasonic curing systems for household or industrial use.

Caraveo-Suarez et al. [111] conducted a study investigating the effects of high-frequency focused ultrasound (HFFU) on beef quality attributes. In this study, triceps brachii muscle samples were treated with HFFU at 2 MHz and 1.5 W/cm^2^ for 0, 10, 20, and 30 min. The results revealed that 30 min of HFFU treatment significantly reduced shear force, indicating improved tenderness, without negatively impacting other quality parameters such as color, pH, drip loss, and WHC. Similarly, Caraveo-Suarez et al. [112] evaluated the application of high-intensity ultrasound (HIU) on longissimus dorsi and triceps brachii muscles from Raramuri Criollo cattle. Their findings indicated that HIU treatment effectively improved meat tenderness without adversely affecting attributes like color, pH, water-holding capacity, and shear force. These results align with the findings of Wu et al. who demonstrated that ultrasonic-assisted low-sodium salt curing combined with sous vide cooking significantly improved the tenderness of beef, making it ideal for elderly dietary applications [113]. Complementarily, Guo et al. [114] applied simultaneous ultrasonic curing on pork (longissimus dorsi) and found that dual- and multi-frequency ultrasound significantly enhanced NaCl diffusion, reduced cooking loss, and improved WHC and texture.

**Figure 7:**
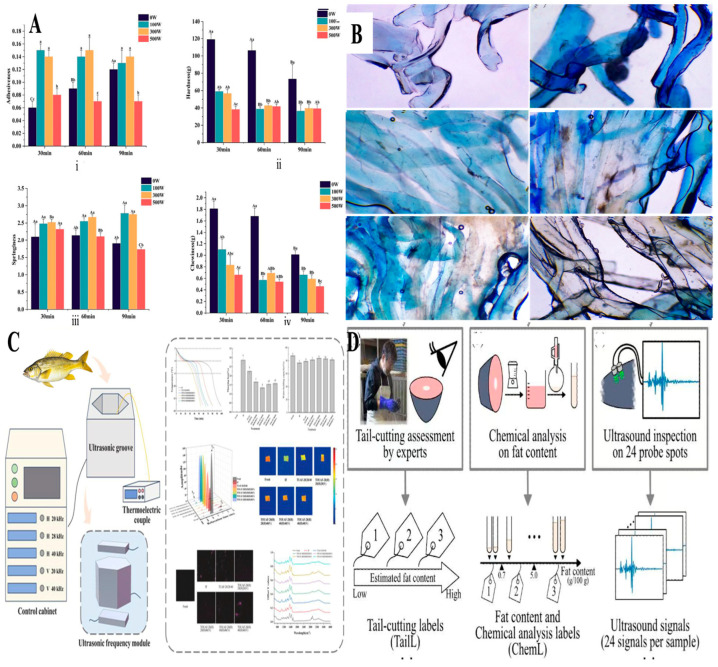
Ultrasound in seafood. (**A**) Effects of ultrasonic treatment on the texture of marinated sea bass fillets. (**i**) Hardness, (**ii**) Efficiency, (**iii**) Springiness, and (**iv**) Chewiness at different treatment times (30, 60, 90 min) and power levels (0 W, 100 W, 200 W). Ultrasound significantly reduced hardness and chewiness, while low-power treatments enhanced adhesiveness and springiness [116]; (**B**) Microstructure of cod samples determined by microscope with a magnification of 4 times. Reproduced with permission from [117]; (**C**) Impact of triple-frequency orthogonal ultrasound-assisted freezing (TOUAF) on the physicochemical properties of large yellow croaker. TOUAF improved freezing rate, water-holding capacity, and protein solubility, while reducing lipid oxidation and protein degradation [118]; (**D**) Evaluation of fat content in albacore using the traditional tail-cutting method, chemical analysis, and ultrasound inspection. Tail-cutting labels (TailL) were compared to chemical and ultrasound measurements for accuracy [119].

Furthermore, in the seafood industry, ultrasound is utilized in various processes, including freezing and thawing. Its application facilitates uniform heat transfer, which shortens processing times and helps preserve the structural integrity of fish tissues. Wang et al. used dual-frequency ultrasound to thaw Larimichthys polyactis and found significant improvements in water-holding capacity, textural uniformity, and protein retention [115]. As summarized in Table 2, ultrasound technology has demonstrated considerable benefits in enhancing texture, quality, and microbial safety across meat and seafood products. For example, chicken treated with pulsed ultrasound in brine exhibits improved juiciness and extended shelf life, while beef and pork experience enhanced tenderness and better fat distribution. In seafood products, ultrasound has been shown to preserve freshness, reduce lipid oxidation, and enhance firmness. Bai et al. [116] investigated the impact of ultrasonic treatment on the texture of marinated sea bass fillets. Compared to static marination, ultrasound significantly reduced hardness and chewiness, while low-power treatments (100–300 W) enhanced adhesiveness and springiness (Figure 7A). These improvements were linked to ultrasound-induced disruption of muscle fibers and improved water-holding capacity. However, excessive power and duration negatively affected texture due to protein denaturation. Dong et al. [117] evaluated the effect of high-intensity ultrasound (HIU) on the physicochemical properties and in vitro digestibility of Atlantic cod (*Gadus morhua*). HIU treatments (0–60 min) enhanced TAC, TFC, TPC, and peptide content by up to 7.28%, 3.00%, 32.43%, and 18.93%, respectively, while improving in vitro protein digestibility by 12.24%. Although total protein content decreased, structural changes observed via color attributes electron microscopy confirmed protein modification (Figure 7B). Furthermore, Yang et al. [118] studied triple-frequency orthogonal ultrasound-assisted freezing (TOUAF) on large yellow croaker (*Larimichthys crocea*) (Figure 7C). TOUAF (20/28 kHz horizontal + 40 kHz vertical) improved freezing rate, boosted water-holding capacity (83.68%), and reduced water migration. It also lowered lipid oxidation (TBA: 0.084 mg MDA/kg) and protein degradation (TVB-N: 9.65 mg N/100 g), while enhancing protein solubility and preserving structural integrity. In addition, Yagi et al. [119] evaluated the accuracy of the traditional tail-cutting method for assessing albacore fat content by comparing it with chemical analysis and ultrasound inspection (Figure 7D). The tail-cutting method showed low accuracy (70.0% and 51.9% across two companies). In contrast, ultrasound inspection, especially when combined with machine learning, achieved 84.2% accuracy, outperforming tail-cutting (73.6%).

Collectively, these findings emphasize ultrasound’s effectiveness in optimizing meat quality, extending product shelf life, and ensuring microbial safety. Moreover, ultrasound-assisted extraction of bioactive compounds from fish by-products has emerged as a sustainable approach for utilizing waste materials. When combined with other technologies, such as heat treatments, ultrasound has demonstrated synergistic effects that further enhance processing outcomes. For instance, combining ultrasound with mild heat can improve microbial inactivation efficiency without compromising the sensory qualities of meat and fish products [120]. In summary, the integration of ultrasound technology in meat and seafood processing represents a promising and innovative avenue for improving product safety, enhancing quality attributes, and streamlining processing efficiency.

**Table 2:** Ultrasound in assessing the texture of meat and seafood.

Meat/Fish	Ultrasound Parameters	Processing Method	Texture	Quality Attributes	Microbial Impact	Nutritional Changes	Observations	References
Chicken	40 kHz, 300 W, 10–40 min	In brine	Improved at 10–20 min, reduced hardness at >40 min	Enhanced juiciness, improved marinade absorption	Reduced bacterial growth, extended shelf life	No major nutrient loss, protein structure preserved	Optimal texture at 10–20 min; longer durations reduced firmness	[121,122]
Beef	20 kHz, 200–400 W, 10–30 min	Direct ultrasound	Increased tenderness	Improved water-holding capacity, better color stability	Significant microbial reduction	Slight increase in protein digestibility	Short treatments improved texture; prolonged exposure led to oversoftening	[123,124]
Pork	22 kHz, 250 W, 5–25 min	Immersion in ultrasound bath	Tenderized, reduced chewiness	Enhanced water retention, better fat distribution	Reduced spoilage bacteria, slower lipid oxidation	Maintained fat integrity, minimal nutrient loss	Best results at 15 min; longer times led to excessive softening	[125]
Salmon	25–40 kHz, 150–350 W, 5–20 min	Direct ultrasound	Improved firmness	Reduced lipid oxidation, extended shelf life	Inhibited bacterial growth, delayed spoilage	Retained omega-3 fatty acids	Low-frequency treatments preserved quality and texture	[126]
Cod	30 kHz, 180 W, 10–15 min	In brine	More elastic, improved texture	Enhanced protein gelation, reduced cooking loss	Lower microbial counts, increased freshness duration	Maintained protein quality, slight fat breakdown	10 min optimized texture without compromising firmness	[127]
Lamb	35 kHz, 250 W, 15–30 min	Direct ultrasound	Increased tenderness, softer texture	Improved flavor absorption, reduced oxidation	Pathogen reduction, extended storage stability	Slight protein degradation at higher exposure	15 min ideal for texture; longer treatments risked excessive softening	[128]
Shrimp	40 kHz, 300 W, 10 min	In brine	Firm, crisp texture	Improved shell removal, better water retention	Lower microbial load, reduced spoilage	Maintained protein and mineral content	Short ultrasound treatments improved peeling efficiency and texture	[129]
Goose	25 kHz, 300 W, 15 min	Direct ultrasound	Softer, juicier meat	Better fat distribution, improved marination	Reduced spoilage organisms	Maintained protein content, improved digestibility	12 min treatment enhanced tenderness and flavor retention	[130]
Turkey	25 kHz, 300 W, 15 min	In marinade	Enhanced tenderness	Improved flavorinfusion, juiciness	Decreased microbial load	Preserved protein content	20 min treatment yielded optimal results	[131]
Duck	25 kHz, 220 W, 10–15 min	Direct ultrasound	Softer texture	Better fat rendering, uniform cooking	Reduced spoilage organisms	Maintained nutritional profile	15 min treatment improved tenderness without oversoftening	[132]
Tilapia	40 kHz, 300 W, 5–15 min	In brine	Firmer texture	Enhanced water retention, improved flakiness	Lower bacterial counts	Omega-3 levels preserved	10 min ultrasound-assisted brining improved texture	[133]
Trout	35 kHz, 250 W, 12 min	Direct ultrasound	Improved firmness	Enhanced color retention, reduced lipid oxidation	Inhibited microbial growth	Nutrient content maintained	12 min treatment optimized quality	[134]
Venison	28 kHz, 275 W, 10–20 min	In marinade	Increased tenderness	Better marinade uptake, enhanced flavor	Decreased microbial load	Preserved protein integrity	15 min ultrasound treatment improved texture and flavor	[135]
Goat	30 kHz, 250 W, 15–25 min	Direct ultrasound	Softer texture	Improved juiciness, reduced cooking loss	Reduced spoilage bacteria	Maintained nutritional value	20 min treatment yielded optimal tenderness	[136]
Mackerel	32 kHz, 200 W, 5–15 min	In brine	Enhanced firmness	Improved protein solubility, better texture	Lower microbial counts	Increased essential amino acids	10 min ultrasound treatment enhanced quality	[137]
Herring	28 kHz, 180 W, 10–15 min	Direct ultrasound	Improved texture	Enhanced lipid stability, extended shelf life	Inhibited bacterial proliferation	Omega-3 content preserved	15 min treatment optimized quality	[138]
Rabbit	35 kHz, 240 W, 10–15 min	In marinade	Increased tenderness	Better flavor absorption, juiciness	Decreased microbial load	Preserved protein content	12 min ultrasound-assisted marination improved texture	[139]
Crab	25 kHz, 300 W, 10 min	Direct ultrasound	Improved tenderness	Enhanced flavor extraction	Reduced microbial load	Maintained protein content	Effective in reducing cooking time	[140]
Mussels	22 kHz, 250 W, 5 min	Immersion in ultrasound bath	Firmer texture	Enhanced water retention	Lower microbial counts	Maintained mineral content	Improved shelf life	[141]
Squid	25 kHz, 220 W, 10 min	Direct ultrasound	Softer texture	Improved moisture retention	Reduced spoilage organisms	Preserved protein integrity	Enhanced tenderness	[142]
Clams	30 kHz, 180 W, 8 min	In brine	Improved firmness	Enhanced flavor retention	Decreased microbial load	Maintained nutrient content	Extended shelf life	[143]
Lobster	28 kHz, 250 W, 12 min	Direct ultrasound	Increased tenderness	Better flavor absorption	Reduced spoilage bacteria	Preserved nutritional value	Improved texture and taste	[144]
Crawfish	35 kHz, 200 W, 10 min	In marinade	Softer texture	Enhanced flavor infusion	Decreased microbial load	Maintained nutrient profile	Improved tenderness and flavor	[145]

### 5.3. Processed Dairy Products

One of the primary applications of ultrasound in dairy processing is found in the production of milk-based beverages, cheese, and yogurt. Notably, ultrasound plays a critical role in assessing gelation, curd firmness, and internal structure. The gelation of proteins in products such as yogurt and cheese is influenced by several factors, including pH, temperature, and enzyme activity [146]. Within this context, ultrasound serves multiple functions, including pasteurization, homogenization, and enhancement of emulsification. Ultrasonic waves induce mechanical vibrations that generate microscopic bubbles in the liquid. These bubbles collapse violently in a phenomenon known as cavitation, releasing localized energy that can disrupt cell walls, increase solubility, and alter the structural properties of proteins and fats [147]. For instance, during milk pasteurization, ultrasound can achieve microbial inactivation without significantly altering the nutritional content or sensory characteristics of the milk, a major advantage over conventional thermal pasteurization methods. In fact, studies have demonstrated that ultrasound-assisted pasteurization can more effectively reduce microbial loads while preserving the flavor, color, and nutritional value of milk [148]. This is particularly important for high-end dairy products, where maintaining product integrity is crucial.

Furthermore, ultrasound technology, especially high-intensity ultrasound (HIU), has gained attention in the food industry for its diverse applications, particularly in dairy processing. This non-thermal technology operates using sound waves at frequencies above 20 kHz, inducing physical effects such as cavitation. These effects have been found to significantly improve the texture, quality, and safety of dairy products [149]. Bermudez-Aguirre et al. [150] demonstrated that ultrasound-assisted pasteurization significantly reduced microbial loads without compromising the sensory quality of milk. This method achieves better microbial stability and emulsion stability, even with extended storage periods. In yogurt production, ultrasound enhances curd formation and influences fermentation kinetics, such as pH reduction rate and viscosity development. By altering the microstructure of proteins, ultrasound improves texture and viscosity in yogurt, yielding a smoother and more uniform product. Similarly, Sun et al. [151] observed that ultrasound-treated milk led to reduced fat globule size and positively affected yogurt’s texture and fermentation rate. Parameters such as pH reduction rate and viscosity development were evaluated, with results showing that HIU influenced fermentation kinetics differently. This, in turn, affected the viscosity and texture of the final yogurt product.

**Figure 8:**
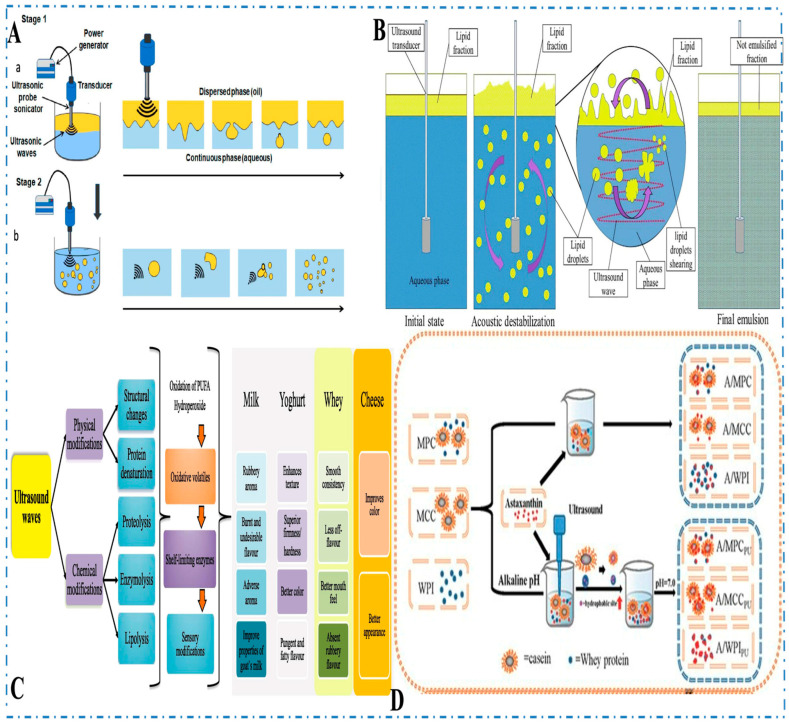
(**A**) Schematic illustration of ultrasonic emulsification. (**a**) The shear forces produced by acoustic cavitation at the interface between the oil and aqueous phases cause the release of large oil droplets (dispersed phase) into the continuous aqueous phase. (**b**) The oil droplets formed in the initial stage are then broken down into smaller droplets due to the shock waves generated during cavitation [156,157]; (**B**) Ultrasound-induced acoustic destabilization of lipid fractions, leading to the formation of lipid droplets and final emulsion. Modified from [158]; (**C**) Ultrasound-induced alterations that result in changes to the sensory characteristics of dairy products; (**D**) Ultrasound-assisted pH-shifting treatment for encapsulating astaxanthin (ASTA) in milk proteins (MPC, MCC, and WPI). The ultrasound improves encapsulation by binding ASTA to the hydrophobic sites of the proteins, enhancing bioavailability and antioxidant capacity which lead to the formation of different nanocomposites: A/MPC, A/MCC, A/WPI, and their PU variants. Adapted from [160].

Additionally, ultrasound has proven effective in enhancing the homogenization of dairy products that require fine emulsions, such as yogurt and cream-based products. Through acoustic cavitation, ultrasound breaks down fat globules into smaller particles, resulting in more uniform and stable emulsions. Ultrasound-treated and homogenized milk typically achieves fat globule sizes ranging from 500 to 900 nm, depending on treatment parameters such as frequency, intensity, and exposure time. For instance, recent studies report average sizes around 870.35 nm in treated samples, compared to approximately 953.39 nm in untreated controls—demonstrating improved emulsification due to ultrasound-induced shear forces and microbubble collapse [152]. This translates to smoother textures and improved mouthfeel key factors for consumer acceptance [153]. In cheese making, ultrasound contributes to moisture control by improving whey drainage and enhancing the firmness and elasticity of the curd, particularly in semi-hard and soft cheeses. These changes are essential for achieving the desired texture and quality [154]. Additionally, Huang et al. [155] explored the effects of ultrasound on the fermentation of skim milk by *Lactobacillus paracasei*. Their study demonstrated that low-intensity ultrasound treatment increased peptide content by 49.5% and viable cell counts by 43.5% compared to untreated samples. This enhancement was attributed to the activation of extracellular enzyme activities by ultrasound. Furthermore, the application of multi-frequency power ultrasound (MFPU) has been studied for its efficacy in protein enzymolysis. Xu et al. [2] reviewed the mechanisms and devices of MFPU, highlighting its role in improving enzymatic hydrolysis processes, which can be beneficial in dairy protein modifications. (Figure 8A) illustrates the emulsification process using high-intensity ultrasound. According to Perdih et al. [156] and Plüisch and Wittemann et al. [157], when two immiscible phases—oil (dispersed phase) and water (continuous phase)—are treated with ultrasonic waves, the shear forces generated during acoustic cavitation promote the rupture of large oil droplets at the oil–water interface (Stage 1). As a result of shock waves from cavitation, these oil droplets are reduced in size, leading to the formation of a finer emulsion with a narrower droplet distribution (Stage 2). In dairy products, the newly formed droplets are further stabilized by emulsifiers such as milk proteins. Kaci et al. [158] also examined the impact of ultrasound on the stability and properties of emulsions (Figure 8B). They found that the cavitation process not only reduces droplet size but also enhances the electrostatic stability of the emulsion. The turbulence generated by the collapsing microbubbles leads to the formation of a charged interface around the droplets, improving their stability against coalescence. This electrostatic stabilization, combined with smaller droplet sizes, results in emulsions that are both more stable and less prone to phase separation. However, more than a decade ago, Chouliara et al. [159] reported that ultrasonication, specifically high-intensity ultrasound (HIU) treatment, failed to extend the shelf life of milk products, as assessed through sensory evaluation. They noted that milk treated with HIU (200 W for 2 min) developed a rubbery aroma and an off-taste described as ‘burnt’ and ‘foreign.’ These undesirable flavor changes have been attributed to protein denaturation, as well as alterations in the physical properties of milk caused by the high heat generated during cavitation. Additionally, some studies suggest that the oxidation of polyunsaturated fatty acid (PUFA) hydroperoxides contributes to the off-flavors in sonicated milk (Figure 8C). This highlights the potential negative impacts of ultrasound treatment on the sensory qualities of milk. Wang et al. [160] found that ultrasound-assisted pH-shifting treatment improved astaxanthin (ASTA) encapsulation, with micellar casein (MCC) achieving the highest encapsulation rate (5.11%), followed by whey protein isolate (WPI) and milk protein concentrate (MPC) (Figure 8D). The resulting nanocomposites enhanced bioavailability, antioxidant capacity, and storage stability, with MCC-encapsulated ASTA showing the best stability over 28 days. Fluorescence and surface hydrophobicity analyses revealed that ASTA occupied the hydrophobic sites on the milk protein. Furthermore, ultrasound helps preserve bioactive compounds in dairy products. For instance, it has been shown to preserve vitamins A and D during milk processing, a significant advantage over conventional thermal treatments that can degrade heat-sensitive nutrients. This preservation of nutrients ensures that dairy products maintain their nutritional value while being processed [161]. Ren et al. [162] confirmed that ultrasound-stabilized corn protein hydrolysates maintained antioxidant properties even after simulated digestion, supporting its role in functional food production. This capability is reflected in improved texture, functionality, and processing efficiency across various dairy products (Table 3). In addition to its impact on product quality, ultrasound can extend the shelf life of dairy products. When combined with packaging techniques like modified atmosphere packaging or high-pressure treatment, ultrasound has been shown to inhibit microbial growth and delay spoilage, thus reducing food waste. For example, ultrasound-assisted processing has improved the storage stability of milk, yogurt, and cheese, allowing for longer shelf life without compromising quality [163]. Products processed with ultrasound remain fresher for longer and display fewer signs of degradation, which contributes to reducing food waste. Additionally, ultrasound can be combined with natural preservatives like essential oils to enhance antimicrobial effects, offering a sustainable alternative to chemical additives.

In summary, the integration of ultrasound technology into dairy processing offers numerous benefits, including enhanced pasteurization, improved homogenization, better texture control, nutrient preservation, and extended shelf life. With consumer demand for such products on the rise, the role of ultrasound in modern dairy processing is poised to become increasingly significant.

**Table 3:** Effects of ultrasound sensory impacts in dairy products.

Dairy Product	Ultrasound Parameters	Observed Effects	Quality Attributes	Sensory Impact	References
Milk	High-intensity ultrasound 20 kHz, 63 °C, 30 min	Reduction in fat globule size, improved homogenization, enhanced microbial inactivation	Improved stability and shelf life	Minimal impact on flavor and taste	[164,165]
Yogurt	High-intensity ultrasound 20 kHz, 31 W, 60 min	Improved rheological properties, enhanced texture, reduced syneresis	Increased viscosity and firmness	Enhanced mouthfeel and consumer acceptability	[166,167]
Cheese	High-intensity ultrasound 20 kHz, 400 W, 10 min	Accelerated ripening, improved texture, enhanced flavor development	Uniform texture and consistent quality	Potential enhancement of flavor profiles	[168]
Whey Protein	High-intensity ultrasound 20 kHz, 400 W, 15 min	Reduction in protein aggregate size, improved solubility, enhanced functional properties	Better emulsification and foaming properties	Neutral impact on sensory characteristics	[169,170]
Ice Cream	High-intensity ultrasound 20 kHz, 150 W, 5 min	Improved emulsification, smoother texture, reduced ice crystal size	Creamier consistency and enhanced overrun	Improved sensory properties, such as creaminess and smoothness	[171]
Butter	High-intensity ultrasound 20 kHz, 200 W, 10 min	Enhanced fat crystallization, improved spreadability, better texture	Softer texture and easier spreadability	Positive consumer perception due to improved spreadability	[172]

## 6. Advantages of Ultrasound Technology

**Figure 9:**
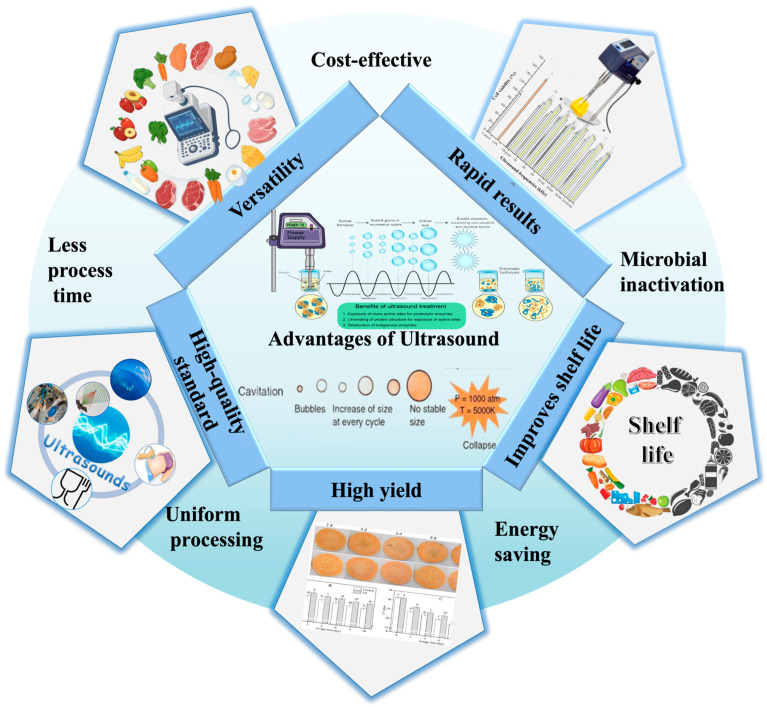
Schematic illustration of the advantages of ultrasound in food texture and quality analysis.

Ultrasound technology has become a cornerstone in modern food processing and quality control due to its unique combination of efficiency, precision, and non-destructive analysis (Figure 9). By leveraging high-frequency sound waves, ultrasound enhances processes such as mixing, tenderization, emulsification, and microbial inactivation—all while preserving heat-sensitive nutrients through low-temperature operation. Additionally, its ability to reduce chemical usage, water consumption, and energy demands makes it a sustainable and cost-effective solution for food industries [173].

### 6.1. Non-Destructive, Real-Time Quality Monitoring

One major benefit of ultrasound is that it provides real-time, non-destructive assessment. Conventional methods such as sampling or testing with another chemical would typically destroy the material, as ultrasound is used to assess intrinsic qualities, like moisture content, texture, and composition, and these attributes can be determined without interacting with the material [174]. Non-destructive sample analysis is extremely useful in bulk commodity production as the production can be monitored continuously to provide high-quality output with minimal waste. For example, Bowler et al. [175] used real-time ultrasound to monitor the gelation process during fermentation of yogurt, allowing them to control texture during production and minimize variability in processing. Meškinytė et al. [176] also validated the use of ultrasound on production lines to assess animal fat content and tenderness while enhancing accuracy in gradings. Real-time feedback further enhances food safety by identifying process deviations early on which can also enhance preventative quality control [173]. Zhang et al. [177] developed a dual-frequency, multi-angle ultrasonic system for soymilk processing that enabled real-time monitoring of protein solubility and emulsifying stability—critical parameters for ensuring product consistency during production. Similarly, Wang et al. [110] employed an ultrasound system for tofu processing that integrated acoustic field monitoring to improve gelation properties without damaging nutritional components. Guo et al. [178] further demonstrated the use of ultrasound for in-line monitoring of milk viscosity and protein aggregation, contributing to more efficient dairy fermentation control. Lastly, Zhang et al. [179] introduced a miniature near-infrared system to monitor structural protein changes in wheat gluten under ultrasound, offering a model for continuous quality control during protein modification. While there may still be accuracy concerns with product variability or changing ambient temperature environments, if it is calibrated properly, ultrasound’s capacity to perform immediate non-destructive sampling increases both productivity and consistency.

### 6.2. Versatility Across Diverse Food Sectors

The versatility of ultrasound encompasses a range of food types, including dairy, meat, fresh produce, and bakery items, in which ultrasound contributes to both processing and quality management [180]. In dairy applications, ultrasound can monitor fat content, moisture, humidity levels, and curd formation of milk-based products [155]. Such consistency is critical for the product development of dairy products such as milk, cheese, and yogurt. For example, Mahmoud et al. [181] presented how ultrasound significantly improved homogenization of milk, resulting in more stable emulsification. Another study further explored ultrasound’s role in dairy-related processes, using dual-frequency ultrasound to enhance the enzymatic hydrolysis of sunflower meal protein, leading to improved enzyme–substrate affinity and reaction efficiency [182]. In another study, Xing et al. [183] applied near-infrared spectroscopy combined with chemometrics to monitor protein fermentation, showing the value of ultrasound-enhanced monitoring tools in solid-state fermentation processes. Moreover, a broader review by Ma et al. [184] outlined how ultrasound-assisted extraction, enzymolysis, and fermentation are pivotal for improving food texture, shelf life, and nutrient retention across various food systems. In the meat sector, ultrasound can determine the tenderness, marbling, and muscle structure for meat products, which aids in the ability to objectively grade and predict the shelf life of the products. Yildiz et al. [185] used ultrasound to assess beef quality based on marbling and texture [186]. For fresh produce, ultrasound assessments can determine levels of ripeness, which aided in monitoring firmness, reduced handling damage, and extended shelf life. While performance is likely to be reduced in analyses with gas or highly complex matrices, the versatility of ultrasound will remain valuable for efficient and consistent production across many food systems.

### 6.3. Minimization of Sample Loss and Waste Reduction

Conventional food testing generates a lot of sample loss because conventional methods can be destructive. Using ultrasound technology in food testing has the unique ability to investigate the integrity of the product without compromising the integrity of the product by conducting the analysis internally [187]. This is particularly useful with higher-than-average costs (e.g., premium meats, artisan cheeses, and rare exotic fruits) or where product quantities are limited. Meškinytė et al. [176] observed that ultrasound could determine beef marbling and tenderness without cutting, which will support real-time (in-line) quality grading. In dairy processing, ultrasound has been able to continuously monitor fat and moisture content without taking the production facility off-line [188]. Additionally, a study on microwave–vacuum drying of tomato slices revealed that precise drying kinetics facilitated by energy-efficient techniques (including ultrasound hybrids) preserved nutrient content and minimized processing loss—particularly of temperature-sensitive compounds like lycopene and ascorbic acid [189]. This will ultimately lead to reduction of raw material waste, a significant reduction in the number of repeated tests, an increase in sustainability efforts, and reduction in other waste. There are limitations to the use of ultrasound technology, including the consideration of signal interpretation in heterogeneous matrices and the initial cost for the equipment. Overall, the benefits of ultrasound technology still present an industry-practical solution to maintain the operational yield of the product while maintaining strict quality control procedures.

## 7. Challenges and Limitations

While ultrasound technology offers numerous advantages—including improved extraction, drying efficiency, and preservation—its wider adoption in the food industry faces several significant challenges. Key barriers include high equipment costs, technical limitations associated with food matrix variability, scalability concerns, and the lack of standardized protocols and regulatory frameworks. This section details these challenges and discusses their implications for broader industrial application.

### 7.1. Technical Constraints in Ultrasound Analysis

Ultrasound’s effectiveness is highly dependent on the physical properties of food matrices, which are inherently diverse and complex [190]. Variations in density, moisture content, texture, and composition can lead to inconsistent ultrasound wave propagation, causing fluctuations in measurement accuracy. For example, dense or highly viscous foods attenuate sound waves more strongly than lighter foods, complicating reliable analysis [191,192]. Another major constraint is the limited penetration depth of ultrasound, especially in thick or multi-layered products like meats and cheeses. In many cases, ultrasound waves can effectively analyze only superficial layers, thus restricting its use for larger samples [2,193]. Additionally, low-resolution imaging limits the detection of fine microstructural features such as cell membranes or delicate tissue layers. Foods with low acoustic impedance contrast (e.g., gels or emulsions) further reduce ultrasound sensitivity. Air pockets, surface irregularities, and coatings like edible films can disrupt sound transmission, causing erroneous results [194,195]. Finally, lack of standardized calibration remains a pressing issue. Current ultrasound instruments require frequent, food-specific calibration to ensure measurement reliability. The absence of universal standards adds complexity and hinders widespread implementation [37,196].

In summary, although ultrasound is a powerful non-destructive tool, improving signal resolution, /penetration capabilities, and calibration standardization is critical for achieving reliable and scalable food applications.

### 7.2. Economic and Scalability Issues

Despite its technical strengths, the high cost of ultrasound equipment remains a major barrier to widespread adoption, especially for small and medium-sized enterprises [197]. Industrial ultrasound systems—comprising sophisticated transducers, power units, and control systems—require substantial capital investment, often without guaranteed immediate returns. Beyond acquisition, maintenance and operational costs further compound the economic burden. Components like transducers are prone to degradation and require regular replacement, while continuous operations demand significant energy consumption [198]. For instance, Zhou et al. [32] highlighted that the energy demands of multi-frequency systems remain a critical factor in cost–benefit assessments for industrial adoption. In fermentation and bioprocessing, ultrasound has shown promise for improving mass transfer and product yield. However, Li et al. [199] examined rare edible fungi fermentation and concluded that, while ultrasound can shorten fermentation time, equipment costs and calibration complexity hinder widespread use. Scaling ultrasound to large-volume food processing also presents technical hurdles. Ultrasound waves are typically effective only over small areas; thus, large-scale operations require multiple transducers or multi-stage processing setups, raising costs and complexity [200]. Ensuring uniform ultrasound treatment across bulk volumes remains challenging, especially in pasteurization, drying, or extraction processes [150]. Achieving consistent ultrasound treatment across food types remains a persistent obstacle due to variability in acoustic properties. Qian et al. [37] emphasized this in the context of enzymatic hydrolysis, where fine-tuned control is required to avoid under- or overtreatment, especially at scale. Additionally, the lack of standardized processing protocols forces producers to tailor ultrasound setups for different foods, leading to inconsistent practices and increased training requirements [180]. In comparison, other emerging non-thermal technologies like cold atmospheric plasma (CAP) and irradiation face similar but more pronounced challenges. Both require high-cost infrastructure and specialized operations, making them less feasible for small-scale applications. CAP struggles with uniform plasma distribution on irregular food surfaces, and irradiation often results in uneven dose application in heterogeneous products. Additionally, CAP lacks harmonized regulatory approval, while irradiation is frequently met with consumer resistance due to labeling and safety perceptions [201]. Sensory changes during storage, such as surface discoloration or flavor degradation, further limit their acceptance. Ultrasound, by contrast, offers a more scalable, cost-effective, and consumer-friendly alternative, reinforcing its potential as a leading non-thermal processing solution.

### 7.3. Standardization and Protocol Development

The absence of universal standards for ultrasound application severely hampers reproducibility and cross-industry adoption [202]. Because ultrasound effectiveness depends heavily on food-specific properties (e.g., density, moisture, texture), tailored protocols are essential to achieve consistent outcomes [6]. Standard protocols must define critical operational parameters: frequency, intensity, treatment time, and transducer configurations for specific food matrices [203]. For example, ultrasound settings optimized for juice pasteurization would differ considerably from those for extracting bioactive compounds from fruit peels. However, heterogeneity in food matrices, equipment variations between manufacturers, and inconsistent operational environments make standardization extremely challenging [204]. Real-time quality control and adaptability must be built into standardized frameworks to accommodate natural variability. Moreover, global regulatory frameworks are still underdeveloped. Agencies like the FDA and EFSA need to establish safety standards, labeling guidelines, and usage regulations for ultrasound-treated food products [205]. Successful standardization will require a coordinated effort among food scientists, engineers, industry leaders, and regulatory bodies, focusing on: comprehensive research into optimal processing conditions; validation across a range of food systems; and formal training programs for operators and manufacturers.

Conclusively, standardized, validated ultrasound protocols are urgently needed to ensure reproducible, safe, and efficient food processing practices, paving the way for mainstream adoption.

## 8. Innovations and Future Directions

Ultrasound technology in food processing is rapidly evolving, driven by innovations aimed at improving efficiency, sustainability, and product quality. Recent developments include the creation of more powerful and energy-efficient transducers, enabling deeper penetration and enhanced consistency in large-scale applications. Researchers are also exploring the integration of ultrasound with other technologies, such as high-pressure processing and microwave treatments, to boost preservation and extraction of bioactive compounds. Furthermore, the use of ultrasound for real-time, non-destructive quality monitoring is gaining attention, offering promising opportunities for improved process control. Looking ahead, optimizing ultrasound parameters for diverse food types, scaling systems for industrial production, and establishing standardized protocols will be crucial for broad adoption. As research advances and accessibility increases, ultrasound is poised to play an even more significant role in sustainable and efficient food processing.

### 8.1. Integration with Advanced Technologies

The integration of ultrasound with advanced food technologies is transforming multiple aspects of food production. High-frequency sound waves generate mechanical vibrations that improve extraction efficiency, enhance food preservation, and accelerate production without compromising quality. One major application is ultrasound-assisted extraction (UAE), which significantly increases the yield of valuable compounds such as essential oils, antioxidants, and flavors by disrupting plant cell walls [206]. UAE reduces processing time and minimizes solvent use, supporting environmentally sustainable extraction processes [207]. In preservation, high-intensity ultrasound (HIU) offers a non-thermal pasteurization method that inactivates microorganisms without applying high temperatures, thus maintaining flavor, texture, and nutritional integrity. Similarly, ultrasound improves emulsification and homogenization processes in dairy, beverages, and sauces by reducing particle size and improving stability [208]. Beyond processing, acoustic sensing enables real-time monitoring of attributes like texture and moisture content, aiding in quality assurance and minimizing waste [209]. Recent advancements have integrated artificial intelligence (AI) with ultrasound systems to enhance data interpretation, automate quality control, and optimize processing parameters. Machine learning algorithms can classify ultrasound signals, detect anomalies in real time, and predict product quality based on acoustic signatures. These AI-driven frameworks are particularly valuable in non-destructive testing of heterogeneous foods, offering scalable solutions for automation in industrial settings. Integrating AI with ultrasound holds promise for improving accuracy, reducing operator dependence, and facilitating smart food manufacturing [210].

### 8.2. Portable and Handheld Ultrasound Devices

The development of portable and handheld ultrasound devices is revolutionizing quality control in the food industry. Compact, user-friendly designs now enable real-time, non-destructive assessment of food quality across production settings. In fruit production, handheld ultrasound devices evaluate firmness and ripeness in crops like avocados, tomatoes, and melons, helping optimize harvest timing and reducing postharvest losses [185]. Similarly, in the meat industry, portable ultrasound assesses fat content and marbling, essential for grading premium cuts and maintaining quality consistency. In food safety, handheld devices detect internal defects, foreign materials, and spoilage indicators in dairy, seafood, and other perishables. Their portability allows quality checks at farms, packing facilities, and distribution centers, offering flexibility across the supply chain. Furthermore, the integration of advanced software facilitates instant data analysis and decision making [211]. These developments strengthen traceability, reduce waste, and enhance operational efficiency, positioning handheld ultrasound as a critical tool for modern food production and distribution.

### 8.3. Expanding Applications in Novel Foods

Ultrasound is increasingly applied in the development of novel foods, particularly in plant-based and functional food sectors. In plant-based protein production, ultrasound-assisted extraction boosts protein, oil, and antioxidant yields from raw materials by breaking down cell walls more efficiently than conventional methods. This improves both nutritional value and production efficiency [212]. Ultrasound also enhances the textural qualities of alternative products. In plant-based meats, it aligns plant proteins to replicate the chewiness and mouthfeel of animal-based meats. Similarly, in dairy alternatives, ultrasound refines textures to create smoother, creamier products. Beyond texture, ultrasound plays a key role in encapsulating sensitive bioactive compounds such as probiotics, vitamins, and omega-3s, protecting them from degradation and enhancing bioavailability [213]. Its non-thermal nature preserves heat-sensitive nutrients during pasteurization and extends shelf life without compromising quality. Additionally, ultrasound contributes to sustainability by maximizing raw material utilization and reducing food waste, particularly during the processing of fruits and vegetables [214]. A study by Khan et al. [215] further reinforces these findings by exploring the role of ultrasound in improving the structural and functional properties of plant-based food ingredients. The researchers demonstrated that ultrasound not only enhances extraction efficiency but also tailors protein structure to improve solubility and digestibility—critical factors for developing realistic meat analogues. The study also showed that ultrasound-assisted emulsification led to greater stability and more effective delivery of bioactive compounds such as polyphenols and omega-3s. Moreover, ultrasound was found to reduce energy usage and waste during processing, aligning with the broader goals of sustainability and clean label food innovation. Altogether, ultrasound offers powerful tools for advancing the production of healthier, more sustainable, and higher-quality food options that meet evolving consumer demands.

## 9. Conclusions

Ultrasound technology has established itself as a critical tool in modern food science for the non-destructive evaluation of texture and quality. Its unique ability to assess structural and mechanical properties—such as firmness, density, elasticity, and internal defects—makes it highly versatile across a broad range of food matrices, including dairy, meat, fruit, and processed products. Low-intensity ultrasound is primarily applied in quality assessment, enabling precise, non-invasive measurements, while high-intensity ultrasound is increasingly utilized for its capacity to induce beneficial microstructural changes, thereby modifying food texture during processing. Compared to traditional analytical methods, ultrasound offers several advantages: rapid data acquisition, minimal sample preparation, and suitability for both solid and liquid foods. These attributes position it as a valuable technique for real-time monitoring and in-line process control.

Looking ahead, the integration of ultrasound with other non-destructive technologies—such as hyperspectral imaging and nuclear magnetic resonance (NMR)—alongside advancements in artificial intelligence and machine learning, is expected to significantly enhance the accuracy, adaptability, and cost-efficiency of quality control systems. The development of portable, miniaturized sensors will also facilitate on-site, real-time evaluation, thereby reducing reliance on centralized laboratory infrastructure and improving responsiveness in quality assurance workflows. Moreover, as global emphasis on sustainability and resource optimization intensifies, non-destructive analysis (NDA) methods will become increasingly essential. Automation and robotics will further support the seamless integration of NDA technologies into smart processing environments, enabling consistent product quality with minimal human intervention. In conclusion, ultrasound and its integration with advanced analytical platforms represent a promising frontier for the future of food texture and quality evaluation. Continued innovation in this area will be crucial to meeting the evolving demands of the food industry, particularly in achieving sustainability, efficiency, and precision in food production and quality management.

## Figures and Tables

**Table 1:** Ultrasound-assisted osmotic dehydration (UAOD) used as a pretreatment in fruits and vegetables.

Fruits/Vegetables	Objective	Ultrasound Conditions	Findings and Quality Effects	References
Strawberry	Explore the efficacy of ultrasound-assisted osmotic dehydration in enhancing water diffusivity and reducing drying time in strawberries, using varied sucrose solution concentrations and treatment times to assess impacts on drying dynamics and fruit structure.	Treatment duration: 10, 20, 30, 45 min; Frequency: 25 kHz; Temperature: 30 °C; Power: 60 W.	Quality potentially enhanced due to improved texture and reduced microbial load, though effects on taste not specified.	[77,78]
	Evaluate the effectiveness of ultrasonic-assisted osmotic dehydration (UAOD) in enhancing strawberry drying rates and quality by varying ultrasonic power and osmotic solutes. The aim is to optimize ultrasound use to improve drying efficiency and outcomes.	Ultrasonic Power: 180 W, 240 W, 300 W; Osmotic solutes: Sucrose, trehalose, sucrose and maltodextrin combination.	UAOD improved texture and nutritional quality; low-hygroscopicity sugars improved drying outcomes.	[79]
Pomegranate	Enhance the efficiency of osmotic dehydration in arils through ultrasound pretreatment, evaluating the effects on moisture removal rates and solute gain.	Frequencies: 25 kHz, 40 kHz; Treatment intervals: Varying.	Ultrasound increased water loss (2-fold at 25 kHz, 2.7-fold at 40 kHz) and solute gain during osmotic dehydration.	[80]
Carrot	Describe the mechanisms by which ultrasound pretreatment enhances the drying and rehydration processes of carrot slices, correlating the effects with structural changes and drying temperatures.	Treatment duration: 30 and 60 min; Frequency: 25 kHz; Power: 41 W/L.	Ultrasound-enhanced drying led to faster rehydration rates and improved process efficiency.	[81,82]
Pineapple	Explore the effects of ultrasound application during both osmotic dehydration and convective drying on the drying kinetics of pineapple, examining the impact on mass transfer and drying rate.	Pretreatment: 20 or 40 min, 55.5 kW/m^3^, 40 kHz; Drying: 21.8 kHz, 31 kW/m^3^.	Enhanced drying rates through ultrasound application suggest improved process efficiency, though specific quality impacts need further study.	[83]
Unripe Banana	Investigate the effects of ultrasound and pulsed-vacuum pretreatments on drying kinetics, diffusivity, and energy efficiency in producing unripe banana flour.	Ultrasound: 20 and 25 min, 35 kHz; combined with pulsed vacuum and air drying at 50 °C and 60 °C.	Ultrasound application preserved resistant starch content, improving the functional quality of unripe banana flour and reducing energy costs compared to conventional processes.	[84,85]
Okra	Examine the impact of ultrasonic combined vacuum pretreatment (UVP) on the drying efficiency and physicochemical properties of okra, optimizing conditions via response surface methodology.	Ultrasound Power: 250 W; Sample thickness: 0.5 cm; Duration: 15 min.	UVP-treated okra showed improved color, bioactive substances, antioxidant activity, and rehydration potential, outperforming control samples.	[86,87]
Persimmon	Assess the impact of ultrasound-assisted osmotic dehydration prior to convective drying on drying behavior and quality properties of persimmon.	Frequency: 35 kHz; Duration: 10, 20, 30 min; 30 °C, 45° Brix and 70° Brix sucrose solutions.	Ultrasound treatment maintained phenolic content and caused minimal color change, preserving quality while enhancing drying efficiency.	[88]
Onions	Investigate the effects of ultrasound and blanching pretreatments on the retention of bioactive compounds and antioxidant activity in onions during drying.	Frequency: 20 kHz; Amplitude levels: 24.4–61 mm; Duration: 1, 3, 5 min.	Ultrasound-treated onions showed higher retention of bioactive compounds and similar color change compared to blanched samples, suggesting it as an alternative to blanching.	[89]
Blackberries	Investigate the effects of airborne and contact ultrasound on the air-drying performances of blackberries, focusing on energy consumption and retention of bioactive compounds.	20 kHz ultrasound probe; Airborne and contact ultrasound methods.	Contact ultrasound yielded blackberries with higher anthocyanin and organic acid content, suggesting superior quality compared to airborne ultrasound.	[90]
White Cabbage	Study the influences of surface contacting ultrasound on the drying kinetics and quality of white cabbage.	Surface contacting ultrasound: 492.3 and 1131.1 W/m^2^; Temperature: 60 °C.	While blanching led to higher vitamin C retention, contacting ultrasound did not alleviate the losses of other phenolics and glucosinolates despite shorter drying times.	[91]
Nectarine Slices	Investigate the effect of ultrasound pretreatment and temperature on the quality and thermodynamic properties of drying nectarine slices in a hot air dryer.	Duration: 0, 10, 20, 40 min; Temperature levels: 50, 60, 75 °C.	The highest shrinkage and color change occurred at 75 °C in control samples. Ultrasound resulted in greater retention of quality attributes at varying temperatures.	[92]
Ginger Slices	Examine the effects of various pretreatment methods, including ethanol and ultrasound, on the drying process and quality of catalytic-infrared-dried ginger slices.	Ultrasound: 40 kHz, 300 W; Volumetric power: 60 W/L; Duration: 15 min; Ethanol: 75% (*v*/*v*).	Ethanol + US-pretreated ginger retained higher amounts of bioactive compounds, although rehydration ratio and gingerol content slightly decreased.	[93]
Black Chokeberries	Explore the mechanisms of water transport and metabolic pathways of polyphenols in black chokeberries dried by sequential calcium pretreatment and ultrasonic/microwave drying.	Ultrasound: 20 kHz, 300 W; Diameter: 5.5 cm; Inserted into a convective dryer.	Enhanced retention of phenolic content; the modified diffusional model provided insights into cellular water dynamics and polyphenol metabolism under hybrid drying technologies.	[94]
Red Peppers	Examine the effects of ultrasound-assisted vacuum (USV) drying on the drying rate and quality parameters of red peppers at various temperatures.	Ultrasound-assisted vacuum drying at temperatures of 45, 55, 65, and 75 °C.	USV drying resulted in significant reductions in yeast and mold counts, suggesting enhanced microbial safety without compromising bioactive compound stability.	[95]
Sour Cherries	Measure the impacts of gum-based coatings and sonication before drying on various quality parameters of sour cherries.	Ultrasound: 40 kHz, 150 W; at 25 °C for 12 min.	Coatings significantly enhanced bioactive compound retention and reduced color changes and shrinkage, with basil seed gum showing the best results.	[96]
Cauliflower	Investigate the effects of single- and dual-frequency ultrasound washing, combined with additives, on the microbial reduction and shelf life of freshly cut cauliflower.	Hexagonal ultrasonic washing tank: 20, 28, 40 kHz; Power densities: 30, 40, 50 W/L; Modes: Pulsed, sweep; Additives: ZA, TS, ET.	Use of zinc acetate as a washing solution particularly benefited the quality maintenance of cauliflower during storage, enhancing bioactive compound content and reducing microbial load.	[97]
Wolfberry	Investigate the effects of ultrasonic frequency, power, irradiation height, and temperature on the drying characteristics and quality of wolfberry by ultrasonic-assisted far-infrared drying.	Frequencies: 40 kHz; Power: 24 W; Temperature and irradiation height varied.	Highest antioxidantactivity and total flavonoid content observed in optimized conditions, enhancing quality preservation and accelerating the drying process.	[98,99]
Kiwifruit	Optimize ultrasonic treatment combined with sodium hypochlorite on kiwifruit to enhance microbial control and maintain textural quality.	Ultrasonic intensity: 184–368 W/cm^2^; Temperature: 25–40 °C; Treatment time: 8–15 min; Solvent concentration: 30–60 ppm.	Enhanced textural quality and extended shelf life of whole and fresh cut kiwifruits, showing potential for chitosan-coated fresh cut fruits.	[100]
Fresh Vegetables	Enhance the antimicrobial efficiency of slightly acidic electrolyzed water (SAEW) through ultrasonication and water wash treatments for sanitizing fresh vegetables.	SAEW parameters: pH 5.2–5.5, oxidation reduction potential 500–600 mV, available chlorine concentration 21–22 mg/L; Ultrasonication: 3 min; Water wash: 150 rpm, 1 min.	The optimized treatment effectively reduced yeast, mold, and bacterial counts on fresh vegetables, suggesting a potential for improving produce safety.	[101,102]
Plum Fruit	Investigate the individual and combined effects of aqueous chlorine dioxide and ultrasonic treatments on the postharvest storage quality of plum fruit.	Aqueous chlorine dioxide: 40 mg/L for 10 min; Ultrasound: 100 W for 10 min; Modes: Simultaneous (one-step), sequential (two-step).	No detectable chemical residues were found in treated samples using the one-step mode, ensuring safety for consumers. The treatment maintained content of total flavonoids, ascorbic acid, reducing sugars, and titratable acids.	[103]
Pakchoi	Evaluate the use of ultrasonic treatment in combination with modified atmosphere packaging to preserve pakchoi.	Frequency: 30 kHz; Duration: 5, 10, 15 min; MAP: 5% O_2_ + 10% CO_2_ + 85% N_2_.	This method effectively reduced peroxidase and polyphenol oxidase activities, indicating enhanced preservation of pakchoi during storage.	[104]
